# Mobilization of the AbGRI4 resistance island in *Acinetobacter baumannii*: IS*26* action and homologous recombination both contribute

**DOI:** 10.1128/spectrum.00389-26

**Published:** 2026-04-17

**Authors:** Christopher J. Harmer, Johanna J. Kenyon, Ruth M. Hall

**Affiliations:** 1 School of Life and Environmental Sciences, The University of Sydney 98483, Sydney, New South Wales, Australia; 2 Sydney Infectious Diseases Institute, The University of Sydney 442308 , Sydney, New South Wales, Australia; 3 School of Pharmacy and Medical Sciences, Health Group, Griffith University, Gold Coast Campus 548144 , Southport, Queensland, Australia; Zhejiang University School of Medicine Sir Run Run Shaw Hospital, Hangzhou, Zhejiang, China

**Keywords:** *Acinetobacter baumannii*, global clone 2, carbapenem resistance, AbGRI4, aminoglycoside resistance

## Abstract

Carbapenem-resistant *Acinetobacter baumannii* isolate HUMC1 (USA, 2009), which is hypervirulent and extensively antibiotic resistant, has been used in many experimental studies. The complete sequence of the chromosome and two plasmids was determined, and the causes of the resistance phenotype were investigated. HUMC1 (ST2:ST1839:KL22:OCL3) is an unusual global clone 2 (GC2) isolate that lacks an AbGRI1 type resistance island interrupting the *comM* gene. However, a version of AbGRI2, which is present together with AbGRI1 in the oldest known GC2 isolate, and versions of AbGRI3 and the less common AbGRI4 were found in the chromosome. The *oxa23* carbapenem resistance gene is carried by two copies of Tn*2006*. The remaining resistance gene complement is distributed among the three AbGRI, which each carry a *sul1* type class 1 integron remnant bounded by IS*26* with a gene cassette conferring resistance to useful aminoglycosides; *aacC1* (gentamicin) in AbGRI2, *aacA4* (gentamicin and tobramycin) in AbGRI3, and *aadB* (gentamicin and tobramycin) in AbGRI4. The *armA* gene (amikacin, gentamicin, tobramycin) was also in AbGRI3. The likely source of AbGRI4 was traced to an ST406 lineage. It was imported into HUMC1 or its ancestor, surrounded by a 23 Kbp ST406 segment that is not normally present in GC2 and incorporated into the chromosome by recombination in homologous flanking regions. The segment carrying AbGRI4 has also been acquired by the ST499 and ST229 lineages. The HUMC1 features highlight the importance of class 1 integrons, insertion sequences IS2*6* and ISAba1, and homologous recombination in the evolution of resistance in this species.

**IMPORTANCE:**

HUMC1 is a carbapenem-resistant *Acinetobacter baumannii* isolate belonging to the dominant globally disseminated GC2 clonal complex that is often used in experimental studies as a highly virulent and extensively antibiotic-resistant representative of the species. However, its resistance gene complement had not been examined. The complete genome reported here revealed that HUMC1 carries multiple genes conferring resistance to aminoglycoside antibiotics within chromosomally located resistance islands, including AbGRI4, which is not widespread in GC2 isolates. AbGRI4 appears to have been acquired from an ST406 type co-circulating with GC2 in the USA. The ST499 clonal group, also co-circulating in the USA, has also acquired AbGRI4 from the same source. HUMC1 is also unusual because it lacks an AbGRI1 resistance island found in the *comM* gene in most current GC2 isolates and in the earliest currently available GC2 isolates from Europe. Hence, it may represent a form that is a precursor of those isolates.

## INTRODUCTION

*Acinetobacter baumannii* is an opportunistic pathogen and a leading cause of difficult-to-treat resistant infections in healthcare settings, particularly intensive care units where antibiotic use is high (1–3). The species thrives in hospital environments and resists eradication. Its untreatable status is largely attributable to the presence of large genomic islands, each containing several different antibiotic resistance determinants that were acquired prior to the availability of carbapenems, the current treatment of choice, and the subsequent acquisition of a carbapenem resistance determinant (4). These pre-existing characteristics, coupled with carbapenem resistance genes acquired later, have led to the development of extensively antibiotic-resistant lineages.

*A. baumannii*, global clone 2 (GC2) has emerged as one of the most clinically significant clonal complexes, and members have been linked to numerous hospital outbreaks worldwide. They are frequently associated with resistance to last-line therapies, such as carbapenems, compounding the difficulty of clinical management (1, 5). Although reports often focus primarily on carbapenem resistance, GC2 isolates are usually extensively resistant because they carry a large suite of acquired genes conferring resistance to many different antibiotics. Among these, resistance to aminoglycosides is particularly problematic, limiting alternative treatment options.

**Fig 1:**
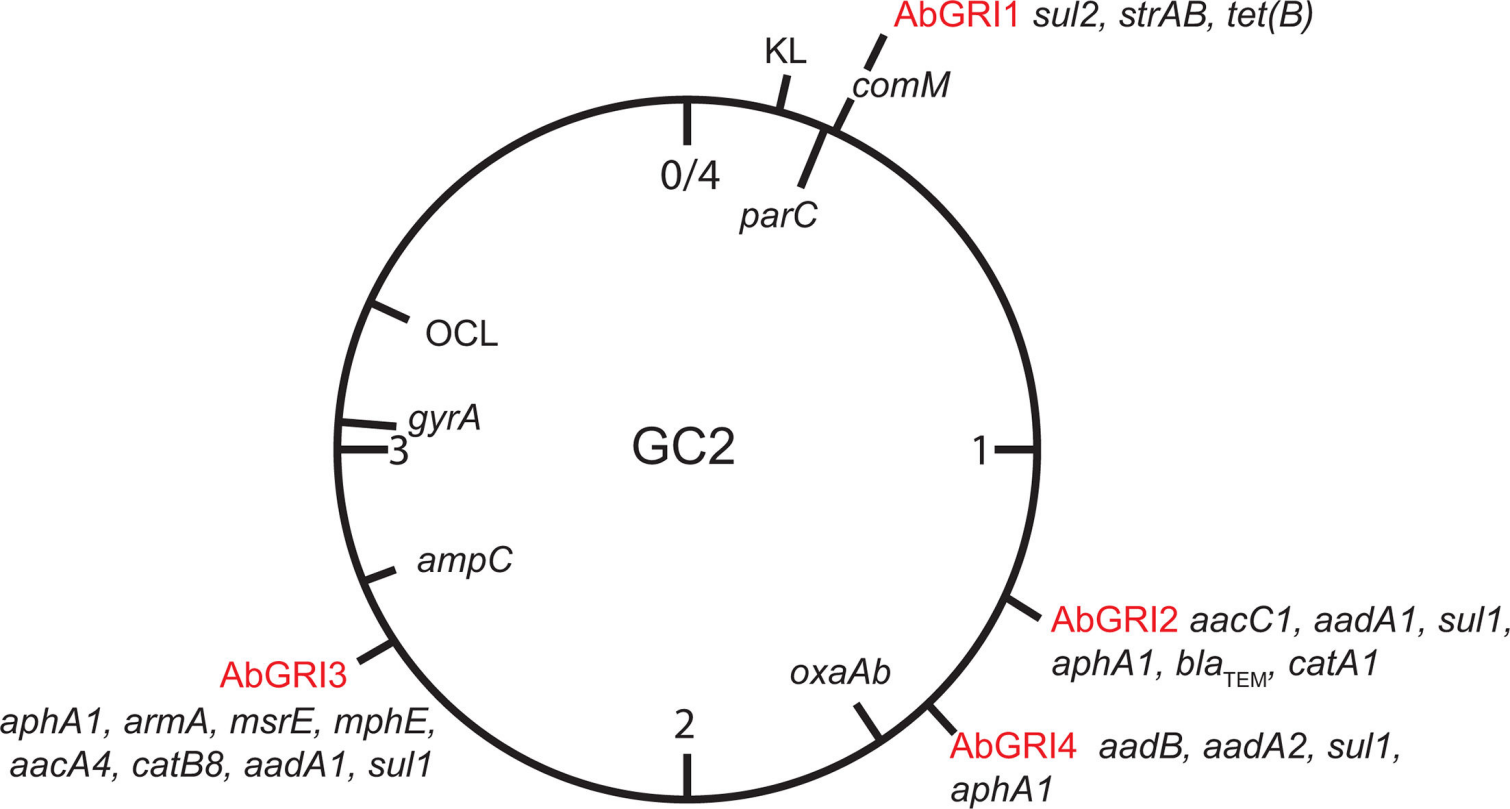
Schematic representation of the complete *A. baumannii* GC2 chromosome. The four main antibiotic resistance islands (AbGRI1, AbGRI2, AbGRI3, and AbGRI4) are marked, with their typical antibiotic resistance gene content listed. The capsule (KL) and outer core (OCL) loci, *ampC*, *oxaAb*, *gyrA,* and *parC* genes are also marked.

The pre-2000 GC2 isolates A320 (RUH134), recovered in 1982 in the Netherlands, and F46, recovered in 1999 in Australia, do not include a carbapenemase resistance gene, and most or all of the acquired resistance genes found in them are clustered in two islands found at specific locations in the chromosome (6, 7). These genomic resistance islands (GRI) are now routinely known as AbGRI1 (*A. baumannii* Genomic Resistance Island 1) and AbGRI2 (6, 8, 9). A third resistance island, AbGRI3, also found at a conserved location, has clearly been acquired more recently (10) but is found in many modern-day GC2 isolates. The fact that the locations of these three GRI (Fig. 1) are conserved indicates that each of them has been acquired only once and thereafter inherited vertically as the GC2 clone expanded. However, significant variation occurs within each GRI as resistance genes are lost and gained (7, 10–12), and, in the case of AbGRI2 and AbGRI3, which were brought in by IS*26* (6, 10, 13), in some isolates, all of the resistance genes have been lost. Nonetheless, in these cases, a single IS*26* remains, revealing that the GRI had been present in that lineage. In other cases, deletions mediated by IS*26* have removed backbone DNA on one or both sides of the GRI (10, 12).

More recently, a fourth chromosomal resistance island, AbGRI4, was detected in several GC2 isolates from the United States (14, 15). This island harbors resistance genes, including *aadB* (tobramycin and gentamicin), *aadA2* (streptomycin and spectinomycin)*, sul1* (sulfonamides), and in one case, namely the version found in HUMC1, an *aphA1* (kanamycin and neomycin) gene. AbGRI4 is surrounded by copies of IS*26* and flanked by an 8 bp target site duplication (TSD), suggesting that, as for the more extensively characterized AbGRI2 and AbGRI3, IS*26* is responsible for incorporating AbGRI4 into the *A. baumannii* chromosome. However, genome sequence data available in 2020 did not allow a clear understanding of the origins and evolution of this GRI to be developed.

In this study, we report the complete genome sequence of the AbGRI4-containing carbapenem-resistant GC2 isolate HUMC1, which was recovered from a case of bacteremia following pneumonia in the United States in 2009 (16). HUMC1 has been used in many experimental studies, e.g., references 17–20, and is known to be resistant to most antibiotics (21). Here, we examine the resistance gene content and GRI configurations in the HUMC1 chromosome. We also track the origin of AbGRI4 via a detailed comparative analysis of the presence and context of AbGRI4 across a large, diverse collection of complete genomes. By characterizing the structure, context, and distribution of AbGRI4, we aim to better understand its role in shaping the resistance landscape of *A. baumannii* and its contribution to the ongoing evolution of multidrug resistance within GC2 and other clinically relevant lineages.

## RESULTS

### Complete genome of HUMC1

The carbapenem-resistant sequence type ST2 (Institute Pasteur scheme) *A. baumannii* isolate is a member of global clone 2 (GC2). HUMC1 has previously been reported as extensively antibiotic resistant, exhibiting resistance to all clinically relevant antibiotics except colistin (21). Here, antibiotic susceptibility testing showed that HUMC1 is resistant to carbapenems, imipenem, meropenem, and doripenem, and to ampicillin and ampicillin-sulbactam, cefotaxime, ceftazidime, cefepime, tazobactam, and ceftriaxone. It was also resistant to fluoroquinolones, ciprofloxacin, and levofloxacin (and nalidixic acid), and to clinically relevant aminoglycosides amikacin, gentamicin, tobramycin, as well as netilmicin, kanamycin, neomycin, streptomycin, and spectinomycin. Resistance to sulfamethoxazole, trimethoprim, tetracycline, and doxycycline was also recorded.

**Fig 2:**
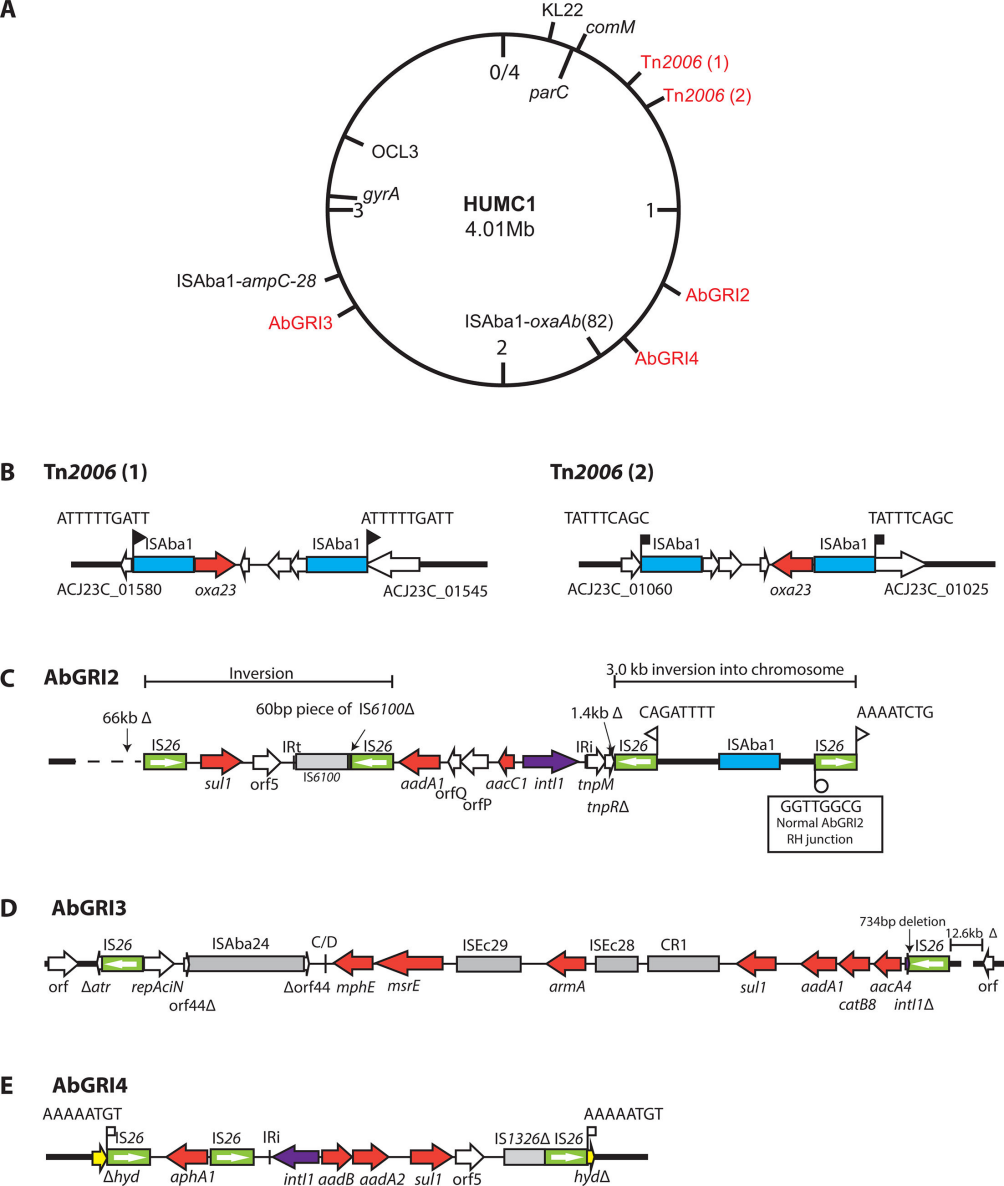
HUMC1, transposons, and antibiotic resistance islands. (**A**) HUMC1 chromosome. The locations of two copies of Tn*2006*, AbGRI2, AbGRI3, and AbGRI4 are marked in red. Configuration of the two copies of Tn*2006* (**B**), AbGRI2 (**C**), AbGRI3 (**D**), and AbGRI4 (**E**). Horizontal arrows show the extent and direction of reading frames with names above and below. The locus tags surrounding the two copies of Tn*2006* are marked below. Antibiotic resistance genes are shaded red. Complete and partial copies of insertion sequences are shown as boxes with names above, with an arrow inside copies of IS*26* to indicate the IS orientation. TSDs are shown as sequences above vertical flags, and inverted repeats are shown as vertical lines.

To enable detailed analysis of resistance islands and other genomic features, the sequence of the complete genome of HUMC1 was determined. Hybrid assembly using Unicycler produced a single circular chromosome of 4,014,688 bp (Fig. 2A; GenBank accession no. CP175646), along with two plasmids: a 29,278 bp r3-T1 plasmid (accession no. CP175645) and a 108,159 bp r3-T9 plasmid (accession no. CP175647). A draft genome of HUMC1 (seven contigs) had been previously determined (GenBank accession no. LQRQ00000000), though the sequence had not been directly reported in a publication. The complete and draft chromosomes differed by just three single-base deletions. HUMC1 belongs to ST1839 (Oxford scheme) and carries the KL22 capsule biosynthesis locus and the OCL3 outer core locus (Fig. 2A). However, the *gtr6* gene of KL22 is interrupted by an ISAba13, and hence, a variant form of the capsular polysaccharide is produced, leading to enhanced virulence (19). This variant CPS form has been designated K3-v1 (22). A total of 20 copies of ISAba1 were found distributed in the chromosome, six of them associated with beta-lactam resistance (see below), but no ISAba125 were detected.

### Carbapenem, cephalosporin, and fluoroquinolone resistance

Analysis of the complete genome revealed that all of the antibiotic resistance genes were located in the chromosome, and the plasmids were not examined further. Resistance to ciprofloxacin and levofloxacin is conferred by mutations resulting in S81L and S84L amino acid substitutions in GyrA and ParC, respectively. A copy of ISAba1 is located in the standard position 9 bp upstream of the *ampC* gene (23) in the orientation that enhances gene expression (Fig. 2A), accounting for resistance to ceftazidime and cefotaxime (24, 25). However, although the region surrounding *ampC* is identical to that in the reference GC2 isolate A320 (GenBank accession no. CP032055), the *ampC-28* allele differs by a single nucleotide and a 3 bp insertion from the *ampC-19* allele found in A320 and typically found in many GC2 strains. These differences lead to a P238R substitution and an additional alanine residue at position 243 in the AmpC-28 protein (between 242 and 243 in AmpC-19).

A copy of ISAba1 is also located 7 bp upstream of the *oxaAb* gene in the orientation that enhances expression, and the 7 bp spacing has been noted in other cases of ISAba1 upstream of *oxaAb* (7, 26). Here, the *oxaAb*(82) allele that differs from the *oxaAb*(66) allele typically found in most GC2 strains by a single nucleotide difference (SND) is present. This causes a single amino acid (aa) change L167V in OxaAb, leading to production of the OXA-82 form, which is one of only a few associated with the ability to confer meropenem resistance when an ISAba1 is present upstream (26, 27). Although this configuration would contribute to carbapenem resistance, two copies of Tn*2006* (Fig. 2B) carrying the *oxa23* carbapenem resistance gene (conferring resistance to imipenem, meropenem, and doripenem) are present (Fig. 2B). Early GC2 isolates like A320 do not include acquired carbapenem resistance genes, and here one of the two Tn*2006* copies (copy 1 in Fig. 2B) was found to be surrounded by an 18 kbp segment that is identical to the corresponding region in global clone 1 (GC1) isolates. Hence, it appears that the *oxa23* gene in Tn*2006* was first acquired at this location by a GC1 isolate and later transferred to HUMC1 from that source. Subsequently, Tn*2006* moved to the second location.

### Location of acquired genes conferring antibiotic resistance

The other acquired antibiotic resistance genes in HUMC1 are found in multiple acquired regions in the chromosome. Notably, whereas in most GC2 isolates the *comM* gene is disrupted by insertion of a complex transposon structure known as an AbGRI1 resistance island, in HUMC1 the *comM* gene remains intact. The resistance genes known to be associated with AbGRI1 [*sul2*, *tetA*(B), *strA,* and *strB*] were not detected, and the source of resistance to tetracycline and doxycycline was not identified. The absence of AbGRI1 suggests that AbGRI2 may have entered the ST2 type genome before AbGRI1.

HUMC1 carries three copies of *sul1* (sulfonamide resistance), two copies of *aadA1,* and one of *aadA2* (streptomycin and spectinomycin). These genes are usually associated with class 1 integrons (28–30) and further gene cassettes containing resistance genes are also present, *aacC1* (gentamicin)*, aacA4* (gentamicin, tobramycin, kanamycin)*, aadB* (gentamicin, tobramycin, kanamycin)*,* and *catB8* (chloramphenicol) (28, 29, 31). The *armA* gene (amikacin, gentamicin, tobramycin, kanamycin) that adds amikacin resistance (32) and the *aphA1* (kanamycin and neomycin) are also present. Finally, the *mphE/msrE* (macrolide resistance) gene pair normally found in a *dif* module (33) is in an incomplete *dif* module that lacks one of the p*dif* sites. These genes are distributed among three genomic resistance islands, AbGRI2 (6, 9), AbGRI3 (10), and AbGRI4 (15), that are each surrounded by copies of IS*26* and found at a specific position in the chromosome (see Fig. 1 and 2A). As the activity of IS*26* often causes deletions or inversions of segments of DNA adjacent to it (reviewed in reference 34), in any individual GC2 isolate, there can be considerable variation in the internal content of these GRIs, and adjacent DNA is also often missing. The specific structure of each GRI in HUMC1 is described below.

### AbGRI2

The ancestral AbGRI2 island is the 32.4 Kbp AbGRI2-0 structure, which is inserted within a hypothetical gene (locus tag A320_01207, in the GC2 reference strain A320 GenBank accession no. CP032055) and surrounded by an 8 bp TSD (5′-CGCCAACT-3′) (6). HUMC1 carries a substantially reduced derivative of AbGRI2-0, as internal IS*26*-mediated deletions and inversions have truncated the island resulting in a residual 10,102 bp structure that retains only three of the original six resistance genes, namely *aacC1*, *aadA1,* and *sul1* (Fig. 2C). Although the right-hand junction of AbGRI2 with the chromosome is retained in HUMC1, it is now located approximately 3 Kbp from the current right-hand boundary of the island due to an IS*26*-mediated inversion. The original left-hand junction is absent, as a large 66 Kbp chromosomal segment has been deleted, also mediated by IS*26*.

### AbGRI3

The prototypical AbGRI3 is an IS*26*-bounded structure, made up of two overlapping IS*26*-bounded pseudo-compound transposons (PCTs), PTn*6179* carrying *aphA1* and PTn*6180* carrying *armA*, *msrE-mphE,* and a class 1 integron remnant that carries *aacA4* (10). The *aacA4* gene encodes the protein form that confers gentamicin, tobramycin, and kanamycin resistance. The location of AbGRI3 is shown in Fig. 1. The AbGRI3 island in HUMC1 is 18,339 bp long (Fig. 2D) and includes only a modified version of PTn*6180* that has lost 734 bp adjacent to the left-hand IS*26* and truncated the *intI1* gene. Hence, this island retains key resistance genes. The left-hand junction is intact, but a second deletion, likely also IS*26*-mediated, has removed the right-hand junction and approximately 12.3 Kbp of adjacent chromosomal DNA and may have removed Tn*6179*.

### AbGRI4 in HUMC1

As noted previously (15), the 10,381 bp AbGRI4 island in HUMC1 (Fig. 2E) interrupts a gene predicted to encode an α/β hydrolase (bases 1,566,939–1,567,540 and 1,577,922–1,578,023 in GenBank accession no. CP175646). It is bounded by directly oriented copies of IS*26* and surrounded by an 8 bp TSD (5′-AAAAATGT-3′), a hallmark of IS*26*-mediated cointegration (34, 35). The AbGRI4 island in HUMC1 (Fig. 2E) differed from the AbGRI4 in other GC2 isolates examined by Chan et al. (15) in that it includes a second IS*26*-bounded segment with the *aphA1* kanamycin and neomycin resistance gene in the central segment. This second novel PCT, which we have designated PTn*7820* and described elsewhere (36), overlaps and shares an IS*26* with the PCT carrying a class 1 integron. The integron includes the *aadB* and *aadA2* gene cassettes along with part of the 3′-conserved segment (3′-CS) containing *sul1*. Here, we found that the complete class integron 5′-conserved segment (5′-CS) is present in AbGRI4 and the 316 bp fragment downstream of *intI1* and beyond IRi is derived from Tn*1696* (Fig. 2D) (see reference 37 for the complete sequence and structure of Tn*1696*).

### GC2 has acquired AbGRI4 and surrounds via homologous recombination

**Fig 3:**
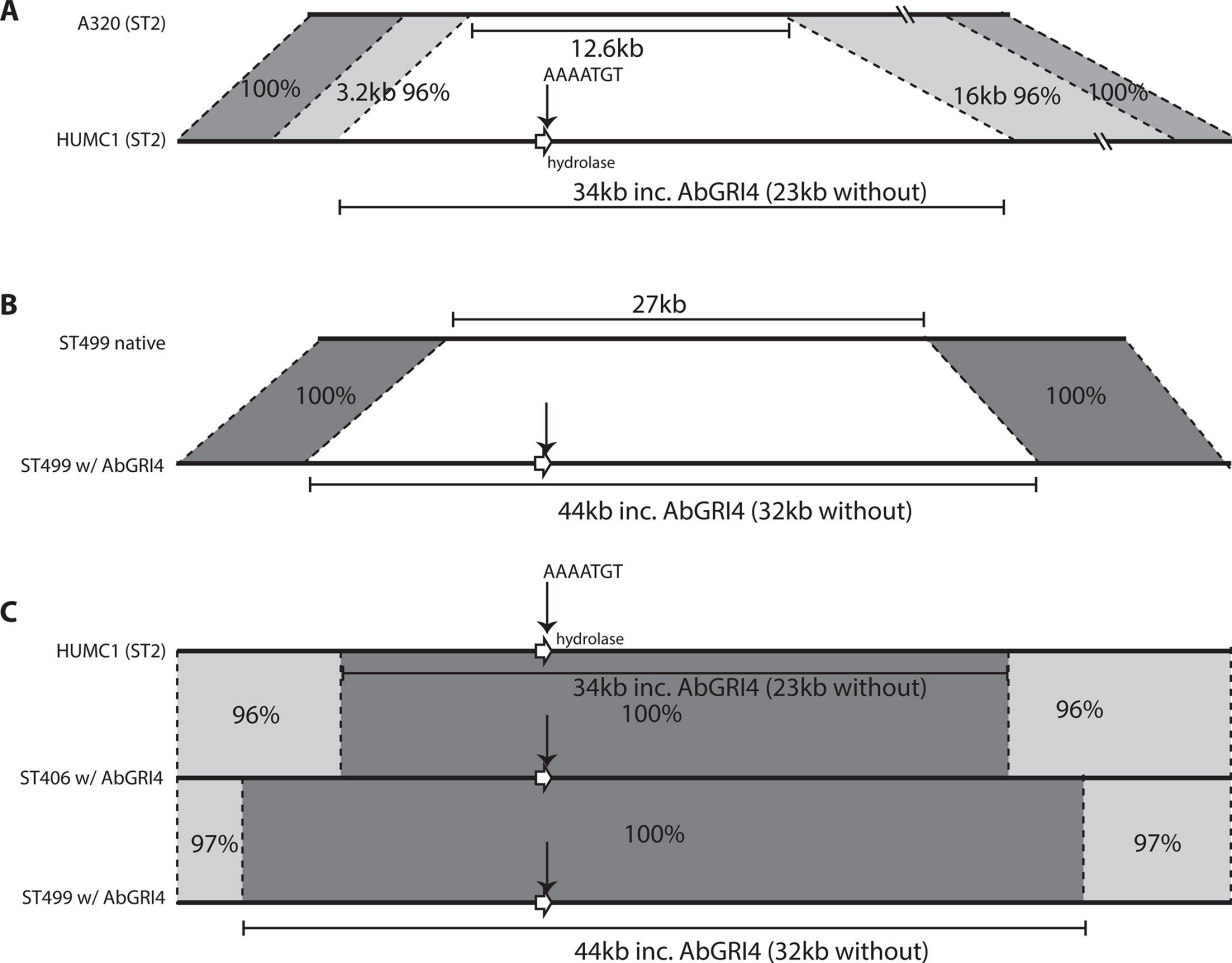
Recombination events introducing AbGRI4 and adjacent chromosomal regions into distinct *Acinetobacter baumannii* lineages. Shaded regions denote high nucleotide identity, with the percentage (%) nucleotide identity marked. (**A**) HUMC1 (ST2/GC2) carrying AbGRI4 compared with the reference GC2 strain A320 (GenBank accession no. CP032055) lacking the AbGRI4 island. (**B**) Representative ST499 strain carrying AbGRI4 (1326581-2, GenBank accession no. CP107597) compared with its AbGRI4-negative counterpart (ARLG6420, GenBank accession no. CP081137). (**C**) Alignment of representative ST2 (HUMC1), ST499 (1326581-2, GenBank accession no. CP107597), and ST406 (19WIARLN021_full, GenBank accession no. CP046552) chromosomes indicates that AbGRI4 was acquired through recombination from a related genomic source.

When AbGRI4 was first characterized in 2020, it was proposed that the island had been acquired by GC2 via horizontal gene transfer or homologous recombination from an unknown source (15). Here, to investigate this further, the unique region surrounding AbGRI4 in the HUMC1 chromosome was first compared to the corresponding region in the GC2 reference genome A320 (CP032055). Alignment of the chromosomal DNA immediately adjacent to either end of AbGRI4 in HUMC1 revealed that it was not present in A320. Indeed, across available complete GC2 genomes, this region was only found when AbGRI4 was present, indicating that this region is not part of the ancestral or standard GC2. Extending the comparison to 30 Kbp on either side of the AbGRI4 insertion site in HUMC1 revealed that a 33.8 Kbp region, comprising AbGRI4-1 (10,381 bp) along with 10.6 Kbp and 12.8 Kbp of flanking DNA on either side, is not present in A320 (Fig. 3A). In addition, an unrelated 12.6 Kbp segment present in A320 (bases 2,457,026–2,469,659 in CP032055) was missing from the HUMC1 chromosome. This replacement likely occurred via homologous recombination within the regions flanking the replaced and replacing segments, as the flanking regions in HUMC1 are closely related to but share only 98% (left, 3.2 Kbp) and 96% (right, 16 Kbp) nucleotide identity with the corresponding A320 sequences (Fig. 3A).

**Fig 4:**
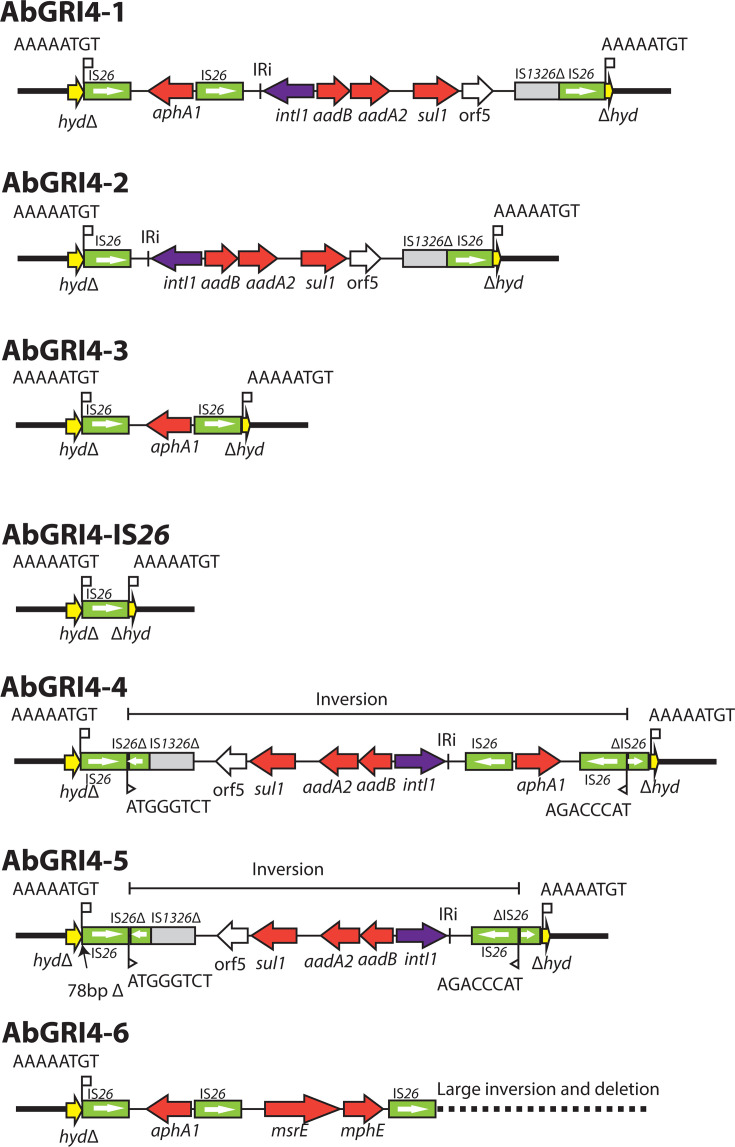
Variants of AbGRI4. Horizontal arrows show the extent and direction of reading frames with names above and below. Antibiotic resistance genes are shaded red. Complete and partial copies of insertion sequences are shown as boxes with names above, with an arrow inside copies of IS*26* to indicate the IS orientation. TSDs are shown as sequences above vertical flags, and inverted repeats are shown as vertical lines. The surrounding chromosomal region is denoted by a thick black line. Inversions or deletions are marked.

The complete genomes of the GC2 isolates ABUH763, ABUH793, and ABUH796 (GenBank accession nos. CP035051, CP035045, and CP035043, respectively), previously reported to contain AbGRI4 (15), were also compared to A320. All three shared evidence of the same recombination patch as seen in HUMC1, although only the integron segment was present in AbGRI4, and IS*26*-mediated deletions on both sides of AbGRI4, 226 bp on the left and 20 Kbp on the right, had occurred (Fig. 4), removing much of the acquired patch and obscuring the original configuration.

### AbGRI4 was similarly acquired by the ST499 clonal complex

A BLAST search against the GenBank non-redundant database (last searched 6/7/2025) detected exact matches to the unique region in HUMC1 that surrounds and accompanies AbGRI4 in several ST406(ST2512) and ST499 complete genomes (Table 1). In the case of ST499 isolates, versions that include all of the unique region and carry a form of AbGRI4 at precisely the same location as found in HUMC1 (i.e., surrounded by the same 8 bp duplication) were found in 10 complete ST499 genomes. These genomes were not available in 2020 when AbGRI4 was first characterized. Further searches (see Materials and Methods for details) retrieved 22 complete ST499 genomes in which AbGRI4 and surrounds were not present. Alignment of the AbGRI4-containing ST499 genomes with a native ST499 genome (e.g., ARLG6420, GenBank accession no. CP081137) showed that the 34 Kbp segment (AbGRI4 plus surrounding 23 Kbp unique region) in HUMC1 is part of a larger 43 Kbp segment (AbGRI4 plus 32 Kbp surrounds) that is not present in the native ST499 chromosome (Fig. 3B). The 43 Kbp segment has replaced a 27 Kbp region of the native chromosome. Hence, the ST499 lineage had also acquired AbGRI4 by homologous recombination in shared flanking regions. This suggested that the ST2 and ST499 lineages may each have acquired AbGRI4 from another source.

**TABLE 1:** Complete genomes containing AbGRI4 or remnants of AbGRI4

Strain	Year	Country	ST (IP)	KL	OCL	AbGRI4 configuration^*a*^	Accession number
Both boundaries intact							
HUMC1	2009	USA	2	22	3	AbGRI4-1	CP175646
19WIARLN022_full	2019	USA	406	9	1	AbGRI4-1	CP046549
2024CK-00130	2024	USA	2512^*b*^	18	3	AbGRI4-1	CP149838
19WIARLN021_full	2019	USA	406	152	1	AbGRI4-2	CP046552
19WIARLN023_full	2019	USA	406	152	1	AbGRI4-2	CP046546
18WIARLN0024	2018	USA	406	152	1	AbGRI4-2	CP043458
19WIARLN011_full	2019	USA	406	152	1	AbGRI4-2	CP046554
1326581-2	2018	USA	499	18	3	AbGRI4-3	CP107597
1326595	2018	USA	499	18	3	AbGRI4-4	CP107603
1326927-2	2018	USA	499	18	3	AbGRI4-4	CP107614
1326924-2	2018	USA	499	18	3	AbGRI4-4	CP107608
1326927-1	2018	USA	499	18	3	AbGRI4-4	CP107612
1326589	2018	USA	499	18	3	AbGRI4-4	CP107601
1326924-3	2018	USA	499	18	3	AbGRI4-4	CP107610
1326527-1	2018	USA	499	18	3	AbGRI4-4	CP107585
1326581-1	2018	USA	499	18	3	IS*26* only	CP107595
1326359	2018	USA	499	18	3	IS*26* only	CP107577
Deletion at left boundary							
2024CK-01444	2024	USA	499	18	3	IS*26* only^*c*^	CP174438
2022CK-00063	2022	USA	499	18	3	AbGRI4-5^*d*^	CP115632
Deletion at right boundary							
AR_0078	Unknown	Unknown	229	17	1	AbGRI4-6^*e*^	CP026761
Lv648	2020	USA	229	17	1	IS*26* only^*f*^	CP169832
1326525-2	2018	USA	499	18	3	AbGRI4-2^*g*^	CP107581
1326525-1	2018	USA	499	18	3	AbGRI4-2	CP107579
1326525-3	2018	USA	499	18	3	AbGRI4-2	CP107583
Deletion at both boundaries							
ABUH793	2015	USA	2	22	3	AbGRI4-2^*h*^	CP035045
ABUH796	2015	USA	2	22	3	AbGRI4-2	CP035043
ABUH763	2015	USA	2	22	3	AbGRI4-2	CP035051
ARLG_6344	2018	USA	2	22	3	AbGRI4-2	CP081139
Lv647	2020	USA	195^*i*^	22	3	AbGRI4-2	CP169828
AB189-VUB	Unknown	Belgium	2	22	3	AbGRI4-2	CP091353
AB222-VUB	Unknown	Belgium	2	22	3	AbGRI4-2	CP091343

^*a*^ See Fig. 4.

^*b*^ SLV of ST406.

^*c*^ Just an IS*26* and IS*26*Δ, no resistance genes. 110 Kbp deletion into chromosome on the left of the island location.

^*d*^ 78 bp deletion into the chromosome on the left of the island location.

^*e*^ *aphA1* transposon and a 2832 IS*26*-bounded fragment from Tn*1548*.

^*f*^ Just an IS*26*, no resistance genes. A large deletion in the chromosome on the right of the island location.

^*g*^ 5 Kbp deletion into the chromosome on the right of the island location.

^*h*^ 226 bp deletion to the left, 20 Kbp deletion to the right of the island location.

^*i*^ SLV of ST2.

### AbGRI4 is also present in the chromosome of ST229 and ST406 isolates

A variant form of AbGRI4 was also found in two ST229 genomes. As noted previously, the content of one of them was different (AbGRI4-6 in Fig. 4) with a PCT carrying a fragment of the *msrE-mphE dif* module (see reference 33 for a description of the *dif* module). A single draft ST229 genome (GenBank accession no. VHHI00000000) lacked both AbGRI4 and surrounds, indicating that again the unique segment surrounding AbGRI4 is not native to this lineage.

The entire novel segment found in ST499 genomes that carry AbGRI4 was also present in six complete ST406 genomes. However, in this case, searches of complete and draft *A. baumannii* genomes for relatives that did not include AbGRI4 did not return any matches. Nonetheless, based on the temporal order of reports of AbGRI4 characteristics in genomes of isolates from US health systems (see Discussion), it seems likely that ST406 was first to acquire AbGRI4 and hence the likely source of the island and its surrounds in ST499 and GC2 isolates.

### Variation within and around AbGRI4 is generated by IS*26*

To investigate the distribution and structural diversity of AbGRI4, complete genomes were screened using two approaches. First, the interrupted hydrolase gene was used to identify genomes with intact left and/or right chromosomal junctions. This approach identified only the 10 ST499 genomes and 6 complete ST406 (or single locus variant) genomes carrying AbGRI4 identified above (Table 1). Among these 16 genomes, five distinct structural configurations were observed (Fig. 4). The complete AbGRI4-1 configuration from HUMC1 with two overlapping PCTs was found in two of the ST406(2512) genomes. Four ST406 genomes contained only the IS*26*-flanked partial class 1 integron (AbGRI4-2) and AbGRI4-3, which contains only the IS*26*-flanked *aphA1* pseudo-compound transposon, was found in one ST499 genome. Finally, two ST499 isolates had only an IS*26* insertion at the chromosomal site. These configurations could have arisen from AbGRI1-1 via homologous recombination within the directly oriented IS*26* copies. The final seven ST499 genomes had the full content of the two overlapping PCTs rearranged by an internal IS*26*-mediated inversion. This configuration (AbGRI4-4 in Fig. 4) has arisen via an attack by the left-hand IS*26* on a site internal to the right-hand IS*26*.

Second, the internal AbGRI4 configuration from HUMC1 was used to detect variants with flanking chromosomal deletions. This identified the two ST229 genomes described above and five ST499 complete genomes that have only one intact boundary (Table 1). The ST449 AbGRI4 structures all displayed complex internal rearrangements (Fig. 4), primarily mediated by IS*26*, as well as deletions extending into the flanking chromosomal DNA on one or both sides of the island. One genome contained the AbGRI4-5 configuration, which is clearly a derivative of AbGRI4-4 that has lost the *aphA1*-containing segment.

A further seven ST2(195) genomes lack the IS*26*-bounded *aphA1* PCT and retain only the IS*26*-flanked partial class 1 integron (AbGRI4-2) but lack DNA at both the left and right chromosomal boundaries in HUMC1. This group includes the three ST2 isolates in which AbGRI4 was first described, plus four further ST2 (or ST2 SLV) genomes. In each case, the structure includes a 226 bp deletion into the chromosome on the left side and a ~20 Kbp deletion on the right. This group includes the only isolates not recovered in the USA, and as the adjacent deletions are identical to those found in the USA isolates, they were likely imported from that source.

### Phylogenetic relationships

**Fig 5:**
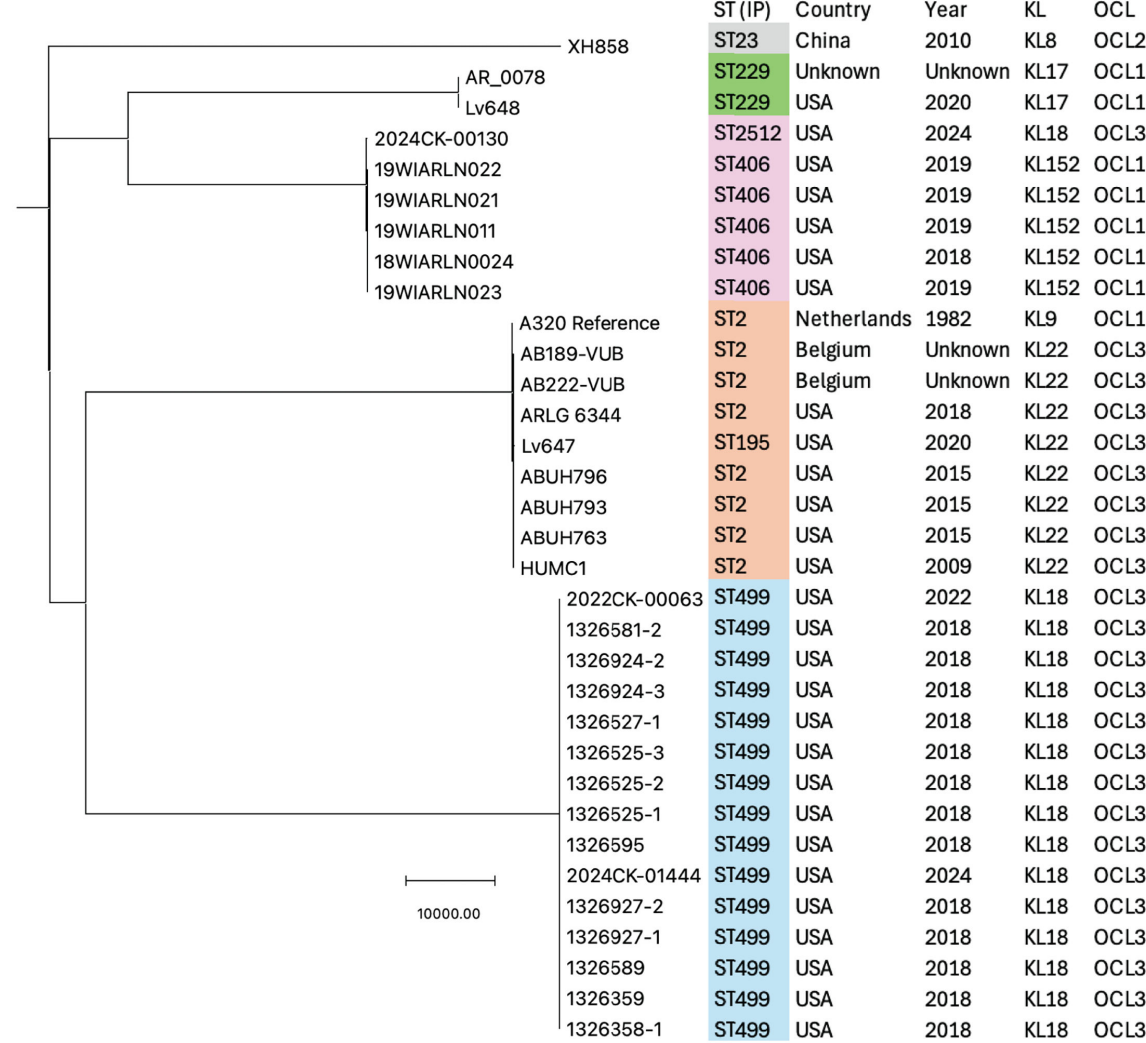
Midpoint-rooted SNP-based phylogeny and key features of the AbGRI4-containing isolates. The ST23 isolate XH858, which contains an uninterrupted hydrolase gene, was used as the out-group. Shared STs (Pasteur scheme) are shaded.

Most of the groups (ST + SLVs) of isolates examined here included ones that carried the same KL and OCL types (e.g., KL22 and OCL3 for the GC2 group). Only the ST406/2512 group exhibited variation (Table 1). To further examine the evolutionary relatedness of the different AbGRI4-containing STs, a midpoint-rooted SNP-based phylogeny was generated using Snippy with the early GC2 isolate A320 (CP032055) as the reference genome (Fig. 5). An ST23 genome (XH858; GenBank accession no. CP014528) that contains the intact hydrolase gene but with different surroundings was included as an outgroup. The genomes of isolates in the different STs each formed a closely related group. The ST499 isolates were the most closely related, with individual pairs of isolates separated by 23–92 SNPs in total. Isolates in the ST2, ST406, and ST229 groups were a little more diverse, separated by up to 432 SNPs. KL switches and large recombination events, combined with separate evolutionary histories, coincide with the major branch points in the tree.

In the course of this analysis, we noticed a large region of about 644 Kbp in the HUMC1 chromosome that is only 98.8% identical to the sequence of A320. This recombination patch extends from position 3,628,107 in the HUMC1 sequence across the origin to position 257,665 and includes a very large number (7,499) of SND. However, as the patch does not span any of the MLST alleles for the Institut Pasteur scheme (it lies between the MLST alleles *rplB* and *rpoB*), the ST does not change.

## DISCUSSION

Two chromosomally-located genomic resistance islands, AbGRI1 and AbGRI2, have been routinely found associated with multiply and extensively resistant GC2 isolates recovered over the past four decades (9–11). AbGRI1 and AbGR2 have been incorporated each at a specific chromosomal location (see Fig. 1) and, although their internal content (including genes that confer resistance to aminoglycosides, tetracyclines, sulfonamides, β-lactams, and other antibiotics) varies, they are stably maintained in their original location throughout the GC2 clonal complex. This shared location is indicative of shared ancestry. Indeed, the presence of AbGRI1 and AbGRI2 in GC2 genomes has been near-universal, and to the best of our knowledge, the ACICU isolate from Italy represents the only well-documented GC2 isolate that lacks AbGRI1, carrying instead an island of the AbaR-type that is usually seen in GC1 isolates (8). HUMC1 reported here represents the first GC2 isolate confirmed to carry no Tn*6022* family transposon (AbGRI1 or AbaR) in *comM*. Previously, AbGRI1 was not detected in a small set of GC2 genomes (38) that may be close relatives of HUMC1(14). This configuration suggests that AbGRI2 was acquired before AbGRI1. In contrast, the additional GC2 relatives listed in Table 1 that also carry AbGRI4 do include AbGRI1. However, how this difference has arisen and whether further AbGRI1-negative examples are represented in the current large collection of GC2 complete genomes was not further investigated.

Though HUMC1 lacks an AbGRI1, it includes two resistance islands that appeared in members of the GC2 complex somewhat later, namely AbGRI3 and AbGRI4. AbGRI3 may have been acquired from another GC2 isolate, as it is located in the expected position (10) in the chromosome. In contrast, it is clear from the sequence comparisons undertaken here that AbGRI4 was formed elsewhere and brought into HUMC1, as well as the ST499 and ST229 isolates from that source. However, the identity of that source cannot be unambiguously identified using the genome data available currently. Nonetheless, a number of studies have shown that ST2, ST499, and ST406 *A. baumannii* isolates were circulating at the same time in the US healthcare system (39–41). In one study, ST406 isolates with reported characteristics that indicate the presence of AbGRI4 (*aadB, aadA2, sul1* genes present together) were found together with ST499 and ST2 isolates that did not include these genes (39). Based on this observation and the comparative sequence analyses reported here, we conclude that the AbGRI4 resistance island likely arose in an ST406 isolate and was acquired by an ST2, an ST499 isolate, and an ST229 isolate, with the ST406 DNA taken up via natural transformation, followed by recombination within the flanking regions of homology. However, a complete or draft genome for an ST406 isolate that lacks AbGRI4 would be valuable to verify this conclusion.

In the AbGRI4-1 variant seen in HUMC1, the two IS*26*-bounded structures overlap, sharing a copy of IS*26*. Though it is possible that they came in together and were incorporated via the copy-in route, a second scenario is also possible, namely that one PCT came in first and the second was acquired via IS*26*-mediated targeted conservative cointegration, a route that has been demonstrated experimentally (34, 35, 42). Loss of IS*26*-bounded segments via homologous recombination between directly oriented copies of IS*26*, followed by acquisition of new segments via the targeted conservative route, can also explain the different PCT found in the ST229 isolates, and accumulation of further PCTs could occur via this route, leaving the location of AbGRI4 as the primary indicator for the presence of this GRI.

It is well known that the internal content of AbGRI1, AbGRI2, and AbGRI3 can vary, and significant variation in the overall content, specifically the resistance gene content, is often observed. In the case of AbGRI2 and AbGRI3, which were incorporated into the chromosome by IS*26* and are bounded by copies of IS*26,* the variation is mainly due to the action of IS*26,* which removes segments or inverts segments that abut the IS ends, either internal to the GRI or adjacent to it. Here, changes of the same type were also found for the IS*26*-bounded AbGRI4. These various IS*26* activities were recently reviewed (34).

Complete genome sequences facilitate the investigation of the location and configuration of clusters of antibiotic resistance genes, particularly those that are associated with insertion sequences, as the multiple IS copies preclude assembly using short-read-only data. Likewise, the presence of multiple class 1 integrons, as seen in HUMC1, leads to fragmentation. As more complete genomes become available, particularly for isolates that, like HUMC1, are used in experimental studies, they will potentially identify structural variation in the chromosome, such as inversions and deletions of chromosomal segments, the locations of IS, and the presence of phage genomes and potentially important genes associated with them. Ultimately, this will underpin a more detailed understanding of the complexities of the epidemiology and evolution of the GC2 clonal complex.

## MATERIALS AND METHODS

### Genome sequencing and assembly

*A. baumannii* HUMC1 was kindly provided by Dr. Brad Spellberg and has been described previously (16, 21). High molecular weight genomic DNA was prepared from an overnight culture of HUMC1 using a CTAB DNA extraction protocol as described previously (43). Long read and short read sequencing was performed by Plasmidsaurus (San Francisco, United States) using Oxford Nanopore Technology (ONT) and an Illumina MiSeq platform, respectively. This generated 24,368 long reads with an average read length of 6,837 bp (41× coverage) and 6,899,636 150 bp paired-end short reads (274× coverage). Read quality was validated using FastQC (https://github.com/s-andrews/fastqc).

The ONT reads were first filtered using Filtlong version 0.2.1 (https://github.com/rrwick/Filtlong) to remove reads shorter than 1,000 bp and to reduce the output to 500 Mbp of long reads. As described previously (7), hybrid ONT-Illumina *de novo* assembly was performed using Unicycler (version 0.5.0) with default settings, and the assemblies were checked for completeness and contamination using CheckM. Protein coding, rRNA, and tRNA genes were annotated using Prokka (version 1.23). Antibiotic resistance genes and the *oxaAb* allele were identified using AMRFinderPlus, and the *ampC* allele was determined using the *ampC* database (44). Resistance regions were identified and annotated manually using an in-house database of standard sequences. Plasmids were identified and typed using the *Acinetobacter* Plasmid Typing Scheme (version 2.0) (45). The PubMLST database was used to determine sequence type (ST) using the Institut Pasteur MLST scheme. Kaptive (46) was used to identify KL and OCL loci for surface polysaccharide types.

### Identification of genomes containing AbGRI4

Complete genomes containing AbGRI4 were identified by performing iterative BLASTn searches of the GenBank non-redundant nucleotide database. In the original description of AbGRI4, it was identified that the island was inserted in an α/β hydrolase gene, hereafter referred to as *hyd*. The interrupted hydrolase gene into which AbGRI4 has been inserted in HUMC1 (bases 1,566,939–1,567,540 and 1,577,922–1,578,023 in GenBank accession no. CP175646) was used as a query to identify genomes that contained an insertion at this characteristic location. The same search identified genomes with an uninterrupted hydrolase gene. To account for IS*26*-mediated deletions that may remove the left and right chromosomal flanks around AbGRI4, a second search was performed using the HUMC1 AbGRI4 sequence as a query. For each genome identified, the resistance genes, ST, KL, OCL, and plasmids were identified as described above for HUMC1. To facilitate examination of genomes according to their ST, 41,951 genome assemblies were downloaded from GenBank on 16 July 2025 by querying the NCBI FPT site (https://ftp.ncbi.nlm.nih.gov/) with the *A. baumannii* TaxID (471). The downloaded genome assemblies were typed using MLST (https://github.com/tseemann/mlst) to assign sequence types in the *A. baumannii* Pasteur scheme.

### Core SNP phylogeny

A recombination-free approach was used to compare *A. baumannii* isolates in this study to determine their evolutionary relationship. Briefly, whole-genome assemblies were mapped to the GC2 reference sequence A320 using Snippy to generate a whole-genome alignment. High-quality variant sites were called using SAMtools v1.3.1.24, with standard-quality filtering. Single-nucleotide differences in recombinant regions were identified and removed using Gubbins v2.1.025 with default parameters, including the default taxa filtering percentage of 25%. A maximum-likelihood phylogenetic tree was inferred from the resulting recombination-filtered alignment using RAxML (v.8) with the GAMMA model and was visualized using MEGA11.

### Antibiotic susceptibility testing

The antibiotic resistance profile of HUMC1 was determined using a calibrated dichotomous sensitivity disk diffusion assay. The following antibiotics were tested: imipenem, meropenem, ampicillin, cefotaxime, ceftazidime, streptomycin, spectinomycin, sulfamethoxazole, tetracycline, trimethoprim, chloramphenicol, florfenicol, kanamycin, neomycin, gentamicin, netilmicin, amikacin, tobramycin, ciprofloxacin, and nalidixic acid. Resistance profiles were interpreted according to the Clinical and Laboratory Standards Institute guidelines.

## Data Availability

The complete genome of HUMC1 has been deposited in GenBank under BioProject PRJNA835507, BioSample accession number SAMN45068614, and nucleotide accession numbers CP175645, CP175646, and CP175647. Illumina and ONT reads are available under the SRA accession numbers listed in the above BioSample. A list of accession numbers of publicly available complete genomes used in this study is provided in Table 1.
